# Galactose receptor-mediated hepatic targeting system: engineering of quinary cationic liposomes for resveratrol delivery against hepatic steatosis†

**DOI:** 10.1039/d5ra02554k

**Published:** 2025-06-11

**Authors:** Zhijie Liang, Jinzhuai Li, Shuying Luo, Shaorong Li, Kun Zhao, Hongmian Jiang, Yisi Ou, Juan Zhong, Lifeng Luo, Huali Huang, Yingying Li

**Affiliations:** a Research Institute of Lanzhou University in Shenzhen Shenzhen 518000 China; b Medical Experimental Center, The Fifth Affiliated Hospital of Guangxi Medical University Nanning 530000 China hualihuang999@126.com; c State Key Laboratory for the Chemistry and Molecular Engineering of Medicinal Resources, School of Chemistry and Pharmaceutical Sciences, Guangxi Normal University Guilin 541004 China; d Departments of Hepatobiliary Surgery, The First Affiliated Hospital of Guangxi Medical University Nanning 530021 China; e Department of Traditional Chinese Medicine, The Reproduction Hospital of Guangxi Zhuang Autonomous Region Nanning 530000 China lyynaturemed@163.com

## Abstract

Resveratrol (RSV), a natural polyphenol with potent antioxidant and anti-inflammatory properties, exhibits significant therapeutic potential for non-alcoholic fatty liver disease (NAFLD) by modulating lipid metabolism, oxidative stress, and inflammatory pathways. Despite its multi-target mechanisms and enhancement of reverse cholesterol transport, clinical translation remains limited by poor bioavailability and inefficient intracellular delivery. Conventional oral administration fails to achieve therapeutic plasma concentrations owing to extensive first-pass metabolism and low solubility. To address these limitations, this study developed galactose (Gal)-modified lipid nanoparticles (Gal-LNPs) to enhance hepatic targeting *via* asialoglycoprotein receptor-mediated endocytosis. These Gal-LNPs demonstrated significantly improved intracellular RSV delivery (3.49-fold uptake *vs.* unmodified LNPs). In NAFLD mouse models, Gal-LNP-RSV reduced hepatic lipid accumulation and serum alanine aminotransferase/aspartate aminotransferase levels by 48.3% and 58.7%/49.3%, respectively, outperforming free RSV in both aspects. These findings underscore the potential of Gal-LNPs as a transformative means of overcoming RSV's pharmacokinetic barriers, enabling the precise activation of intracellular targets while restoring metabolic and redox homeostasis. This work provides a robust framework for developing targeted nanotherapeutics against NAFLD, bridging the divide between preclinical efficacy and clinical application.

## Abstract 2

Engineered quinary cationic liposomes target liver *via* galactose receptors, delivering resveratrol (RSV) to against hepatic steatosis.

## Graphical abstract

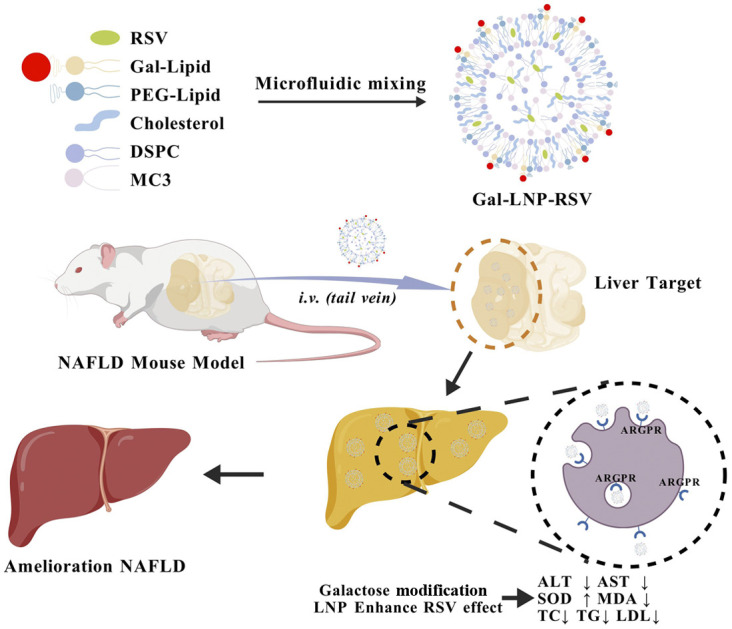

## 1 Introduction

Resveratrol (RSV), a representative natural polyphenol, is widely distributed in medicinal plants, such as grapes (*Vitis vinifera*) and Japanese knotweed (*Polygonum cuspidatum*). Its distinctive chemical structure confers significant antioxidant, anti-inflammatory, and lipid metabolism-regulating pharmacological activities.^1^ In recent years, this compound has attracted considerable attention for its capacity to treat non-alcoholic fatty liver disease (NAFLD), with its mechanism involving the synergistic regulation of hepatic lipid accumulation, oxidative stress, and inflammatory responses.^2,3^ Furthermore, RSV effectively halts NAFLD progression to non-alcoholic steatohepatitis.^4^

RSV's therapeutic mechanisms underlying NAFLD treatment encompass multifaceted regulatory actions, including, but not limited to, the following: (i) the activation of Sirtuin family proteins (notably Sirt1/Sirt3) to enhance mitochondrial β-oxidation and suppress NOD-, LRR- and pyrin domain-containing protein 3 inflammasome activity, thereby reducing hepatic triglyceride (TG) accumulation and pro-inflammatory cytokine levels (*e.g.*, IL-1β, TNF-α, *etc.*);^5^ (ii) modulation of Forkhead box transcription factor class O 3a phosphorylation and nuclear factor kappa-light-chain-enhancer of activated B cells (NF-κB) P65 deacetylation to ameliorate insulin resistance and attenuate hepatocyte steatosis *via* Sirt1-dependent epigenetic pathways;^6^ and (iii) upregulation of low-density lipoprotein receptor (LDLR) and scavenger receptor class B type I expression, which promotes reverse cholesterol transport and very low-density lipoprotein secretion to restore lipid metabolic homeostasis.^7^ While preclinical studies position RSV as a promising therapeutic candidate for NAFLD, multiple randomised controlled trials have demonstrated that oral RSV administration fails to significantly improve serum alanine aminotransferase (ALT)/aspartate aminotransferase (AST) activity or hepatic fat content in patients with NAFLD.^8–10^ The mechanisms underlying this ‘‘preclinical-clinical gap’’ potentially stem from the following critical factors:

First, pharmacokinetic properties substantially limit the bioefficacy of RSV. As highlighted by Milton-Laskibar *et al.* in a systematic review, animal studies typically employ ultra-high RSV doses to achieve hepatic pathological improvements.^11^ In contrast, clinical trials cannot replicate such dosing regimens owing to gastrointestinal tolerability and safety concerns. More importantly, the oral bioavailability of RSV is severely constrained by the first-pass effect. A meta-analysis by Szymkowiak's team revealed that even at a dose of 5000 mg, the plasma concentration of free RSV merely remained at 33.59 ng mL^−1^ (*C*_max_), with ‘time to peak concentration’ (*T*_max_) exhibiting no dose correlation, indicating significant absorption saturation *via* conventional administration routes.^12^ This phenomenon emanates from extensive sulphation/glucuronidation metabolism in intestinal epithelial cells coupled with the inherent low aqueous solubility (∼3 μg mL^−1^) and photolability of RSV, minimizing the quantity of intact drug entering systemic circulation.^13^

Second, insufficient intracellular delivery efficiency compromises the target activation of RSV. The core pharmacological targets (*e.g.*, sirtuins, NF-κB, and protein kinase B [AKT]) of RSV are localised in intracellular compartments.^14,15^ However, free RSV struggles to reach effective concentrations owing to poor membrane permeability and low intracellular accumulation rates. A study by Min JB confirmed that encapsulating RSV with trimethyl chitosan nanoparticles increases cellular drug uptake more than three-fold and significantly upregulates Sirt1 mRNA expression.^16^ Similar delivery strategies have extensively been explored: (i) mitochondriotropic liposomes modified with mitochondrion-targeting peptides generate a five-fold increase in RSV concentration in the mitochondrial matrix, enhancing its oxidative stress regulatory efficacy;^17^ (ii) pH-sensitive poly(lactic-*co*-glycolic acid) nanoparticles achieve selective drug delivery in hepatic stellate cells, suppressing transforming growth factor-beta 1 signalling pathway activation;^18^ and (iii) β-lactoglobulin nanocarriers exploit asialoglycoprotein receptor (ASGPR)-mediated endocytosis to markedly improve RSV intracellular delivery efficiency, thereby overcoming the therapeutic limitations of RSV.^19^

Current research on drug delivery systems primarily focuses on two directions: bioavailability enhancement and tissue/cellular-targeting improvement. To optimise bioavailability, Zhou Min's team developed a spirulina-based floating drug delivery system that prolongs gastric retention by mimicking ‘‘algal bloom’’ phenomena and achieves controlled RSV release *via* ethanol-responsive chitosan/pectin composite membranes, reducing alcohol-induced liver injury markers (ALT and malondialdehyde [MDA]) by 52–67% in C57BL/6 mouse models.^20^ Additionally, amphiphilic block copolymer-based nanomicelles (*e.g.*, methoxy polyethylene glycol-polylactic acid) enhance plasma stability and hepatic metabolic evasion, increasing RSV's oral bioavailability to 4.8-fold that of conventional formulations.^21^ Notably, Min *et al.* utilised an ion-gelation method to prepare chitosan-PEG nanoparticles, which not only elevated RSV's Cmax to 89.3 ng mL^−1^ but also extended intestinal absorption windows through mucoadhesive effects, demonstrating significant ‘area under the curve’ advantages.^16^ Nevertheless, these systems primarily aim to improve systemic exposure, rather than achieving liver-specific enrichment.

In this context, lipid nanoparticles (LNPs) have emerged as a research hotspot owing to their inherent liver-targeting properties and efficient nucleic acid delivery capabilities. Following intravenous injection, apolipoprotein E (ApoE) adsorbed on LNP surfaces specifically binds to hepatocyte LDLRs, triggering clathrin-mediated endocytosis.^22^ Subsequently, the ionisable lipids in LNPs undergo protonation in acidic intracellular environments, inducing membrane destabilisation and cytosolic payload release. Currently, LNP technology has demonstrated ground-breaking efficacy in treating hereditary liver diseases (*e.g.*, transthyretin amyloidosis)^23^ and viral hepatitis.^24^ Nonetheless, the liver-targeting efficiency of current LNPs relies on passive mechanisms. To enhance hepatic accumulation, strategies such as galactose (Gal) ligand modification^25^ and Selective Organ Targeting-LNP technology^26^ have partially overcome passive targeting limitations.

Recent advances in active targeting strategies have provided novel insights into liver-specific LNP delivery. Among these, Gal ligand modification mimics the natural ligand of asialoglycoprotein receptor (ASGPR)-Gal residues, enabling precise hepatocyte targeting. As a C-type lectin receptor abundantly expressed on hepatocyte membranes, ASGPR demonstrates nanomolar-level affinity through its specific recognition and binding to terminal galactose or *N*-acetylgalactosamine (GalNAc) residues. This receptor facilitates efficient intracellular transport of ligand–receptor complexes *via* clathrin-mediated endocytosis, a process that exhibits superior internalization efficiency compared to passive targeting mechanisms.^27^*In vivo* experimental evidence reveals that galactose-modified LNPs achieve remarkedly enhanced hepatic enrichment compared to the unmodified counterparts.^28^ Building upon these findings, Ye *et al.* conducted systematic investigations using various ligand-modified LNP for drug delivery and their result demonstrated that Gal/GalNAc-modified formulations showed markedly higher cellular uptake than unmodified lipid nanoparticles (LNPs), concurrently validating the functional efficacy of ASGPR-mediated endocytosis.^29^ Recent advancements in galactose ligand applications are exemplified by Zhang C. *et al.*'s development of an AA3G LNP system incorporating 2.5% galactose surface modification, achieving a 61-fold increase in hepatic luciferase expression compared with conventional LNPs, thereby validating its superior liver-targeting capacity.^30^ Lu J constructed Gal-functionalised polydisulphide conjugates, leveraging transmembrane thiol-exchange mechanisms to achieve lysosomal escape and the precise intracellular release of biomacromolecules.^31^ Notably, dual-ligand synergistic targeting strategies can transcend the limitations of single-receptor systems; for instance, Yu *et al.* designed Gal-hyaluronic acid cationic solid–lipid nanoparticles that simultaneously target ASGPR and cluster of differentiation 44 receptors, significantly enhancing silibinin uptake in hepatocytes compared with single-ligand systems.^32^ These findings conclusively demonstrate the enhanced hepatic targeting capacity of galactose and reveal its potential for structural modification and expanded therapeutic applications.

Leveraging these advancements, we propose a Gal-modified LNP system (Gal-LNP-RSV) for NAFLD therapy, wherein a novel Gal-lipid conjugate—synthesised *via* a one-step 1-ethyl-3-(3-dimethylaminopropyl)carbodiimide (EDCI)/*N*-hydroxysuccinimide (NHS) coupling reaction between galactosamine and carboxylated polyethylene glycol-distearoylphosphatidylethanolamine (HOOC-PEG-DSPE)—is incorporated as the fifth component to enhance hepatic targeting specificity. By incorporating Gal ligands to achieve ASGPR-mediated active targeting, this strategy is anticipated to overcome the limitations of existing RSV-delivery strategies and provide a novel paradigm for NAFLD therapy.

**Scheme 1:**
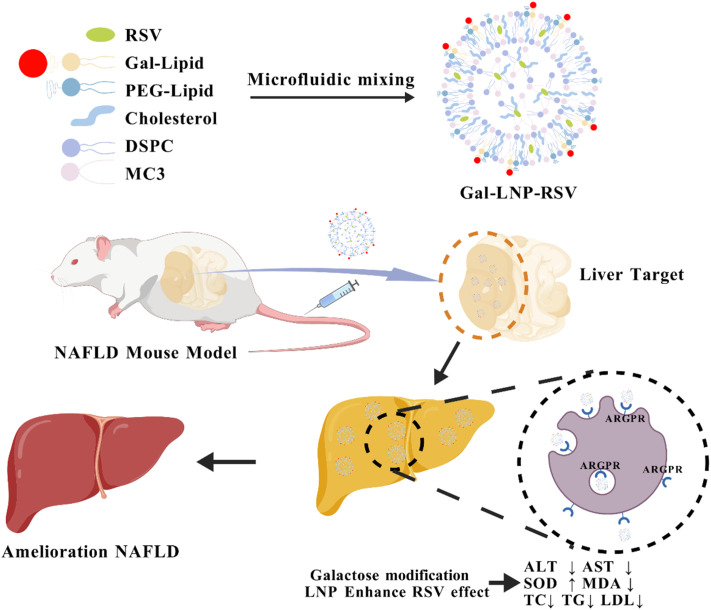


Scheme 1 illustrates the Gal-LNP-RSV preparation process and its liver-targeted delivery mechanism for alleviating fatty liver disease. Through chemical coupling, galactosamine is linked to lipid molecules, forming a targeted ligand. The modified lipids are subsequently mixed with cationic lipids, cholesterol, and other components and assembled into LNPs using microfluidic technology or ethanol injection methods. RSV is encapsulated in the core of the LNPs *via* hydrophobic interactions, and after purification, the Gal-LNP-RSV complex is obtained. The liver-targeting mechanism relies on the specific binding of the Gal ligand to the highly expressed ASGPRs on hepatocyte surfaces, triggering active LNP endocytosis. Once inside the cell, the LNPs release RSV into the acidic lysosomal environment, exerting therapeutic effects on NAFLD.

## 2 Materials and methods

### 2.1 Network pharmacology and molecular docking analysis

The ‘Simplified Molecular Input Line Entry System’ code for RSV was retrieved from the PubChem online database and input into the Swiss Target Prediction website to predict its potential targets. ‘‘Liver injury’’ was searched in the GeneCards, Online Mendelian Inheritance in Man (OMIM), Therapeutic Target Database (TTD), and PharmGKB databases, and ‘‘Nonalcoholic fatty liver disease’’ was searched in the GeneCards, OMIM, and National Center for Biotechnology Information (NCBI) gene databases to obtain liver injury and NAFLD-related genes. The intersection of genes associated with RSV and liver injury, as well as RSV and NAFLD, was obtained using the online Venn diagram tool and plotted. The intersection targets were imported into the Search Tool for the Retrieval of Interacting Genes/Proteins (STRING) database to construct the protein–protein interaction (PPI) network. The top five algorithms of the CytoHubba plugin in Cytoscape 3.9.1—MCC, DMNC, MNC, Degree, and EPC—were used to screen candidate differential genes from the PPI network. The top 10 candidate genes from each algorithm were selected, and overlapping genes across the five algorithms were considered key genes, resulting in five key candidate genes. Gene Ontology (GO) and Kyoto Encyclopedia of Genes and Genomes (KEGG) enrichment analyses were performed on the intersection targets obtained above using the Metascape online platform. The top 10 core pathways were selected based on *P* values, and bubble and bar charts were generated for visualisation using the Microbe Online platform.

Based on the network pharmacology analysis results, the top five core genes associated with NAFLD were selected for molecular docking with RSV. The three-dimensional structure files of RSV and protein structure files of the top five genes related to NAFLD were obtained from the PubChem and PDB databases, respectively. Ligand–receptor molecular docking simulations were conducted using AutoDock software. The complex with the lowest binding energy after docking was selected, the binding sites were determined, and the results were visualised using PyMOL software.

### 2.2 Main materials and reagents

Oleic acid (OA) and palmitic acid (PA) were purchased from Aladdin (USA). RSV was obtained from Aladdin (USA). Oil red O staining solution, Cell Counting Kit-8 (CCK-8), triglyceride (TG), total cholesterol (TC), low-density lipoprotein cholesterol (LDL-C), and high-density lipoprotein cholesterol (HDL-C) assay kits were procured from Beyotime Biotechnology (China). ALT and AST assay kits were acquired from Abcam (UK). DLin-MC3-DMA (MC3), cholesterol, and distearoylphosphatidylcholine (DSPC) were supplied by AVT (China), while DMG-PEG2000 was purchased from JenKem Technology (China). NHS and EDCI were obtained from Aladdin (USA). The HepG2 cell line was sourced from the American Type Culture Collection (USA). Dulbecco's modified Eagle medium (DMEM), other cell culture media, and foetal bovine serum (FBS) were secured from Gibco (USA). Ultrafiltration centrifugal tubes (30 kDa molecular weight cut-off [MWCO]) were acquired from Sartorius (Germany). Finally, 4′,6-diamidino-2-phenylindole dihydrochloride was procured from Thermo Fisher Scientific (USA).

### 2.3 Preparation of Gal-PEG2000-DSPE

Gal-functionalised DSPE-PEG2000 (Gal-PEG2000-DSPE) was synthesised through covalent conjugation. Briefly, 0.8 g of DSPE-PEG2K-COOH was dissolved in 40 mL of dimethylformamide, followed by the dropwise addition of deionised water. To the mixture, 300 mg of EDCI and 200 mg of NHS were added to activate the carboxyl groups. After 120 min of activation, 184 mg of d-galactosamine hydrochloride was added to the reaction system. The reaction was allowed to proceed at room temperature for 48 h with constant stirring. The resulting product was subsequently purified using dialysis (MWCO: 1000 Da) against deionised water for 24 h to remove unreacted reagents and by-products. Finally, the purified Gal-PEG2000-DSPE conjugate was obtained by lyophilisation and stored at −20 °C until use. Structural validation was performed using proton nuclear magnetic resonance (^1^H NMR; 400 MHz, D_2_O).

### 2.4 Preparation of Gal-functionalised RSV LNPs

RSV-encapsulated LNPs (LNP-RSV) were engineered using microfluidic self-assembly technology, enabling precise control over lipid-aqueous phase mixing to achieve monodisperse particle distribution (polydispersity index [PDI] < 0.2) and optimised drug encapsulation efficiency (>85%). The lipid matrix, which comprised MC3, DSPC, cholesterol, and DMG-PEG2000 at a molar ratio of 40 : 10 : 45 : 5, was co-solubilised with RSV in ethanol (total lipid concentration = 10 mg mL^−1^, lipid : drug mass ratio = 10 : 1). The aqueous phase contained 10 mM citrate buffer (pH 7.0, Sigma-Aldrich). Phase convergence was accomplished using a staggered herringbone micromixer at a 3 : 1 flow rate ratio (organic:aqueous), followed by maturation at 25 °C for 10 min. Sterile filtration through 0.22 μm polyvinylidene fluoride membranes (Millipore) yielded stable LNPs, which were subsequently stored at 4 °C. For *in vitro* characterisation, the LNPs were diluted with phosphate-buffered saline (PBS, Corning) to 0.5 ng per μL RSV (ethanol content <5%) and analysed using dynamic light scattering (Zetasizer Nano ZS90, Malvern Panalytical). *In vivo*, grade formulations underwent triple ultrafiltration (30 kDa MWCO, Sartorius) at 3000×*g* (4 °C) for ethanol removal. Gal-functionalised variants (Gal-LNP-RSV) were prepared by adding 2 mol% Gal-PEG2000-DSPE to the base lipid composition. Fluorescent analogues for bioimaging were generated by substituting RSV with cyanine (Cy)3 (*E*_x_/*E*_m_: 552/570 nm) and Cy5 (*E*_x_/*E*_m_: 649/670 nm) probes (Lumiprobe), maintaining identical preparation protocols for *in vitro* and *in vivo* tracking, respectively. For short-term preservation, samples were maintained in 2% (w/v) sucrose solution, while long-term storage protocols employed lyophilized preparations containing blended sucrose and trehalose cryoprotectants.

Drug loading efficiency (LE%) and encapsulation efficiency (EE%) were assessed using high-performance liquid chromatography (Shimadzu, 20A), and DL% was calculated using the following formula: using a standard calibration curve, total (*C*_total_) and free (*C*_free_) drug concentrations were determined. *C*_total_ includes both the encapsulated and unencapsulated drug, while *C*_free_ represents the unencapsulated drug. EE% denotes the proportion of encapsulated drug relative to *C*_total_, while DL% reflects the drug loaded relative to the total Liposome weight. LE% was calculated using eqn (2), where *m*_l_ and *m*_free_ represent the total and free drug, respectively, *n* = 3. The liposome suspensions were diluted with phosphate-buffered saline (PBS, pH 7.40) and subsequently loaded into dialysis cassettes (molecular weight cutoff: 3.5 kDa). The cassettes were immersed in PBS-containing beakers, which were then sealed with aluminum foil and transferred to a constant temperature incubator. The release experiments were conducted under controlled conditions of continuous agitation (60 rpm) at 37 °C. Release efficiency (RE%) is calculated as the ratio of the cumulative drug amount released from nanoparticles (*W*_released_) to the total encapsulated drug (*W*_total_), expressed as a percentage. Quantitative analysis was performed *via* UV-vis spectrophotometry at predetermined time intervals under simulated physiological conditions (PBS, pH 7.4, 37 °C).1
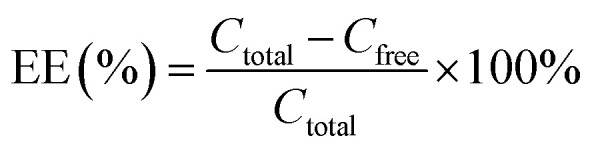
2
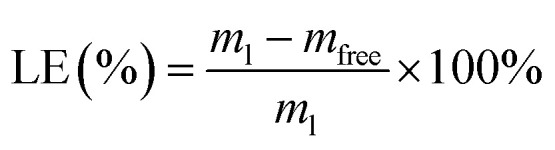
3
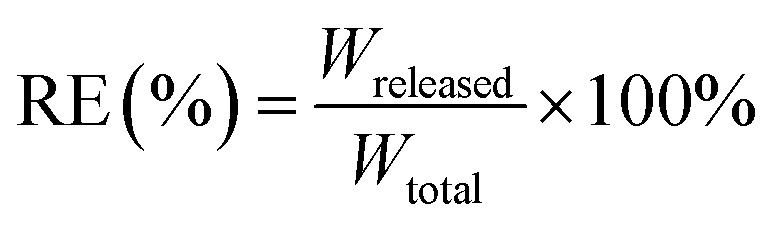


### 2.5 Characterisation approach for LNP-RSV nanocarriers

Transmission electron microscopy (TEM, Tecnai 12; Philips Company, Holland) was used to characterise the morphology of the LNP-RSV nanocarriers. The procedure was as follows: 10 μL of a nanoparticle suspension (1 mg mL^−1^) was negatively stained with 2% phosphotungstic acid solution, subsequently placed on a copper grid, and observed after drying at room temperature. Particle size distribution and zeta potential were measured using a Nano-ZS 90 dynamic light scattering instrument (Marvin Company). The measurements were performed in triplicate on an aqueous dispersion system at a concentration of 0.01 mg mL^−1^ under constant temperature conditions (25 °C). To evaluate the *in vitro* stability of the nanoparticles, the same instrument was used to monitor the Gal-LNP-RSV samples in cell culture medium (containing 10% FBS) over a continuous 12-day period, assessing key parameters such as hydrodynamic diameter and PDI.

### 2.6 Cellular experiments

#### 2.6.1 Cell culture

The HepG2 cell revival and culture protocol involved rapidly thawing frozen cells in a 37 °C water bath, centrifuging to remove cryoprotectant, and seeding into flasks containing high-glucose DMEM. The cells were incubated at 37 °C in a 5% CO_2_ incubator, and cell attachment was observed 24 h post-revival. The medium was periodically refreshed, and the cells were passaged upon reaching 80–90% confluence, ensuring healthy cell growth for subsequent experiments.

#### 2.6.2 CCK-8 experiment

To investigate the biological effects of the drug, the study focused on cellular viability, proliferation, and migration. HepG2 cells were cultured in DMEM containing 10% heat-inactivated FBS and 1% penicillin–streptomycin at 37 °C in a humidified 5% CO_2_ incubator. Thereafter, DMEM supplemented with varying concentrations of the drug was added to 96-well plates (10 000 cells per well). After 24 h incubation with the medium, cell viability was assessed using the CCK-8 assay to evaluate the impact on cellular activity, all tests were conducted with six replicate wells, and the experiment was repeated three times independently.

#### 2.6.3 *In vitro* cellular uptake of nanoparticles

Cells were seeded in a 12-well plate at a density of 2 × 10^5^ cells per well and cultured under 37 °C and 5% CO_2_ conditions for 24 h to facilitate proper adherence. Once the cells had adhered, equal amounts of Cy3-labelled LNP formulations (LNP-Cy3 and Gal-LNP-Cy3 at a concentration of 100 μg mL^−1^) were added to each well, followed by an additional 2 h incubation. To investigate receptor-mediated endocytosis, a Gal inhibition group was established by pre-treating cells with 10 mM Gal for 1 h prior to nanoparticle addition. After incubation, the cells were gently washed three times with pre-cooled PBS to remove any unbound nanoparticles. Subsequently, the cellular uptake of fluorescently labelled nanoparticles was observed and documented using a Nikon fluorescence microscope (NIS, Nikon, Japan). Finally, the uptake mechanism was evaluated by comparing fluorescence intensity between the ‘regular treatment’ and ‘Gal pre-treatment’ groups, thereby assessing the role of the Gal receptor in the endocytic process. Quantitative analysis of the fluorescence signals was performed using ImageJ software, all tests were conducted with six replicate wells, and the experiment was repeated three times independently.

#### 2.6.4 Quantitative analysis of oil red O staining and lipid accumulation in HepG2 cells

Cells were seeded in a 96-well plate at a density of 1 × 10^4^ cells per well and cultured under 37 °C and 5% CO_2_ conditions for 24 h to ensure proper adhesion. Afterwards, the cells were stained and quantitatively analysed using a quantitative oil red O staining kit. Concurrently, TG and TC accumulation levels in HepG2 cells were determined according to the manufacturer's instructions, all tests were conducted with six replicate wells, and the experiment was repeated three times independently.

### 2.7 Animals and diets

Six-week-old female BALB/c mice were obtained from Guangxi Medical University Laboratory Animal Center. The mice were housed in an environmentally controlled room (temperature: 22–24 °C, humidity: 60%) under a 12 h light/dark cycle. After a 1-week acclimation period, the mice were randomly divided into six groups (*n* = 6): a normal chow group (control), a high-fat diet (HFD) group, an ‘HFD combined with free Silymarin *i.v.* group (Silymarin content: 50 mg kg^−1^, once every 3 days), an ‘HFD combined with free RSV *i.v.* group (RSV content: 100 mg kg^−1^, once every 3 days), an ‘HFD combined with LNP-RSV *i.v.* group (RSV content: 100 mg kg^−1^, once every 3 days), and an ‘HFD combined with Gal-LNP-RSV *i.v.* group (RSV content: 100 mg kg^−1^, once every 3 days). After 6 weeks of treatment, the mice were fasted overnight, anesthetised, and subjected to blood sample collection from the vena cava. Their livers were immediately excised, weighed, fixed in 10% phosphate-buffered formalin, embedded in paraffin, and sectioned. The sections were subsequently stained with haematoxylin and eosin (H&E) and oil red O, and observed under an optical microscope to analyze histological structural changes and lipid deposition. In addition, various biochemical parameters in fasting serum were analysed using different assay kits according to the respective manufacturer's instructions. All experimental procedures were performed in accordance with the Guidelines for the Care and Use of Laboratory Animals and approved by the Animal Care and Welfare Committee of Guangxi Medical University (Approval No. 202407014).

### 2.8 *In vivo* biodistribution dynamics of nanoparticles

To investigate the *in vivo* biodistribution of the nanoparticle formulations, the hydrophobic dye Cy5 (excitation/emission: 649/670 nm) was used as a substitute for RSV. Male wild-type BALB/c mice were intravenously injected with 100 μL of three different formulations (*n* = 5): free Cy5, LNP-Cy5, and Gal-LNP-Cy5 nanoparticles (Cy5 dosage: 5 mg kg^−1^). At 2, 4, and 8 h post-injection, the fluorescence intensity of Cy5 was assessed using an *in vivo* imaging system (IVIS® Lumina III; PerkinElmer, USA) to determine its distribution across the body and specific organs over time.

### 2.9 Pharmacokinetic analysis

The pharmacokinetics of LNP-Cy5 nanoparticles (5 mg kg^−1^, intravenous injection) were examined in BALB/c mice (*n* = 5). To assess the *in vivo* metabolism of the different nanoparticle formulations, the hydrophobic dye Cy5 was used as a substitute for RSV. Serum samples were collected at 1, 2, 4, and 8 h post-injection, and the fluorescence intensity of Cy5 was measured at an excitation/emission wavelength of 649/670 nm using a microplate reader.

### 2.10 Biochemical analysis

Cell measurements of TG, TC, LDL-C, and HDL-C were conducted to compare the effects of RSV liposomes and free RSV on various signaling pathways. Additionally, we measured serum levels of TG, TC, LDL-C, HDL-C, AST, and ALT in fasting NAFLD mice using appropriate assay kits. Furthermore, the contents of TG, TC, SOD, and MDA in liver tissues were assessed using biochemical assay kits, *n* = 5.

### 2.11 Statistical analysis

Homogeneity of variances was verified prior to conducting two-way ANOVA with post hoc pairwise comparisons. Data are expressed as mean ± SD. Statistical significance was denoted as follows: *p < 0.05, **p < 0.01, ***p < 0.001 *vs.* control group; #*p* < 0.05, ##*p* < 0.01, ###*p* < 0.01 *vs.* model group; †*p* < 0.05, ††*p* < 0.01, †††*p* < 0.001 *vs.* Silymarin-treated group.

## 3 Results

### 3.1 Multi-target regulation: network pharmacological analysis of RSV in treating NAFLD

**Fig. 1:**
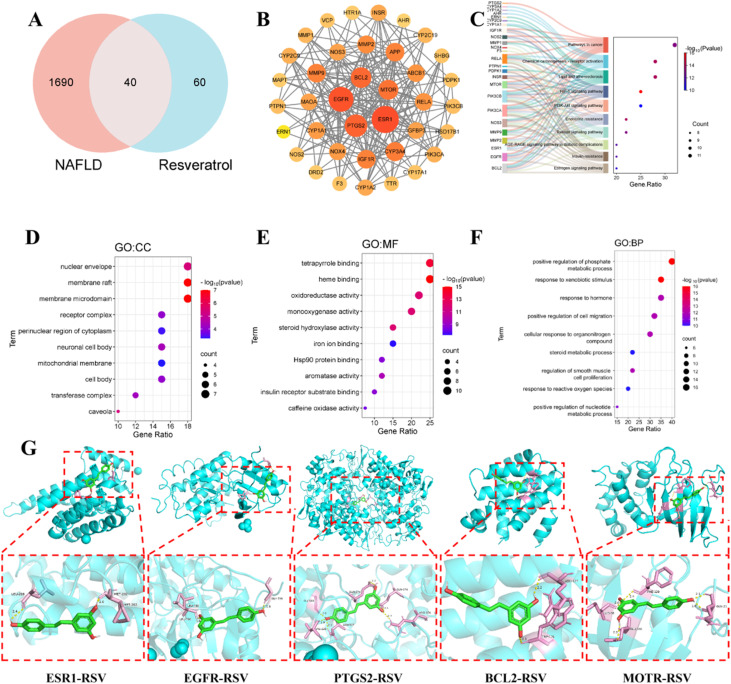
(A) Intersection gene analysis; (B) PPI network; (C) KEGG pathway enrichment analysis; (D) GO : CC functional enrichment analysis; (E) GO : MF functional enrichment analysis; (F) GO : BP functional enrichment analysis; (G) molecular docking of RSV with the top five core targets.

This study employed systems biology approaches to elucidate the molecular mechanisms and signalling pathway regulation of RSV-loaded liposomes in NAFLD. A total of 100 potential RSV targets were identified from PubChem and Swiss Target Prediction databases. Disease-related genes were retrieved from GeneCards, OMIM, NCBI-gene, TTD, and PharmGKB databases, yielding 3258 liver injury-associated genes and 1732 NAFLD-related genes after deduplication. Venn diagram analysis (Fig. 1A) revealed 69 core liver injury-related genes and 40 NAFLD candidate genes overlapping with RSV targets.

The PPI network was constructed using the STRING database, and core modules were identified *via* the CytoHubba topological algorithm in Cytoscape 3.9.1. Key hub genes for liver injury (*BCL2*, *SRC*, *EGFR*, *ESR1*, and *MTOR*) and NAFLD (10 regulators) were prioritised. Multi-level functional annotation *via* the Metascape platform demonstrated significant enrichment of these targets in essential biological processes, such as biological responses to xenobiotic stimuli and the positive regulation of phosphate metabolic processes (GO analysis, Fig. 1D–F). KEGG pathway analysis highlighted central mechanisms involving the phosphoinositide 3-kinase (PI3K)-AKT signalling axis, the hypoxia-inducible factor 1 pathway, lipid metabolism regulation, and atherosclerosis-related pathways (Fig. 1C).

Molecular docking simulations confirmed strong binding affinities between RSV and the top five NAFLD core targets, with binding energies below −5 kcal mol^−1^ (Fig. 1G), indicating favourable interaction stability.

This study is the first to systematically decode RSV's multi-target synergistic mechanism in NAFLD. RSV exerts multidimensional hepatoprotection by modulating apoptosis-related BCL2 pathways, metabolic hub MTOR complexes, and EGFR-mediated cell survival signalling. Notably, most core targets are localised in intracellular compartments, underscoring the necessity of liposomal delivery systems that enhance RSV's intracellular bioavailability. Liposomal encapsulation not only improves drug stability^33^ but also facilitates transmembrane transport *via* membrane fusion mechanisms,^34^ thereby enabling the effective activation of intracellular signalling networks.

### 3.2 Preparation and characterisation of Gal-LNPs

**Fig. 2:**
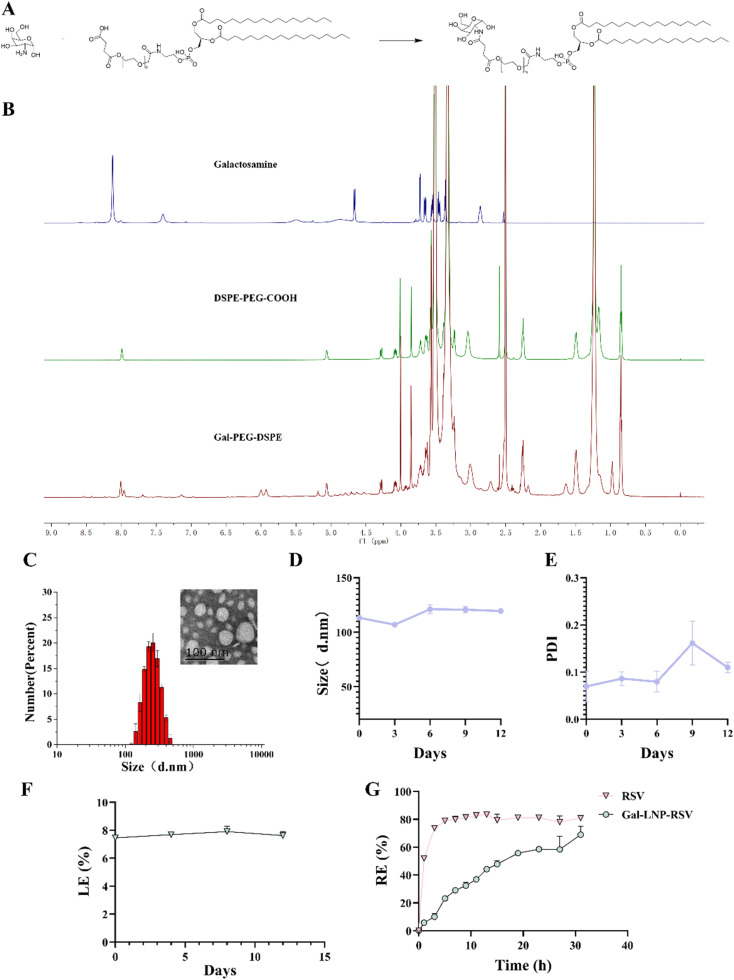
(A) Synthetic route of Gal-PEG-DSPE; (B) ^1^H NMR spectrum; (C) particle size and TEM images of Gal-LNPs, *n* = 3; (D) particle size distribution profiles of Gal-LNPs, *n* = 3; (E) PDI variation curves of Gal-LNPs, *n* = 3; (F) drug loading efficiency (LE%) of lipid nanoparticles across experimental groups; (G) *in vitro* release profiles demonstrating Release Efficiency (RE%) under physiological conditions (PBS, pH 7.4, 37 °C).

To enhance the intracellular delivery efficiency of LNPs, we introduced a novel hepatotropic component by conjugating galactosamine to HOOC-PEG-DSPE *via* EDCI/NHS chemistry, as illustrated in the synthetic route (Fig. 2A). Successful conjugation was confirmed using ^1^H NMR spectroscopy (Fig. 2B), where characteristic peaks of 3–5 ppm indicated Gal incorporation into the lipid structure. Thereafter, Gal-PEG-DSPE was incorporated as a quinary component into LNPs to construct Gal-LNP-RSV. The formulation exhibited excellent colloidal stability, with its particle size remaining stable (100 nm, PDI <0.15) over 12 days under physiological conditions (Fig. 2C–E), demonstrating its potential as a robust drug carrier.

As demonstrated in the study of Hea Ry Oh *et al.*,^36^ a co-delivery LNP system targeting the ASGPR was developed which utilized galactose-modified cationic LNP to encapsulate the doxorubicin and vimentin siRNA, forming nanoparticles with an average diameter of approximately 100 nm for synergistic hepatocellular carcinoma (HCC) therapy, achieving drug encapsulation efficiency of 80.57% with a loading efficiency (LE) of 7.46%. Notably, the *in vitro* release profile demonstrated significantly prolonged drug release kinetics, showing only 58.58% cumulative release within the initial 24-hour period. Comparative parallel experiments further confirmed that galactosylation modification enhanced the sustained-release characteristics of RSV in our study, consistenting with findings by Minhee Kim *et al.*, galactose-functionalized liposomes were shown to substantially prolong drug half-life.^37^

### 3.3 Effects of RSV on lipid metabolism in OA/PA-induced HepG2 cells

**Fig. 3:**
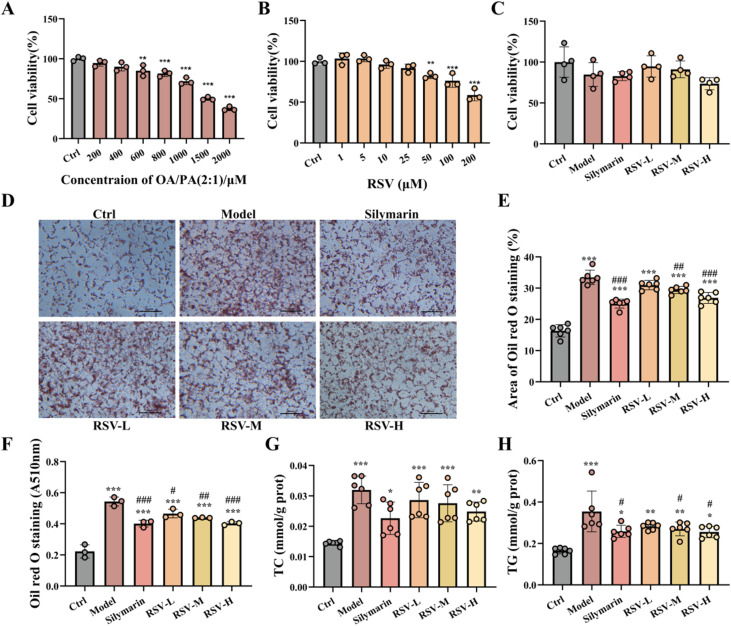
(A) Cell viability test under OA/PA (2 : 1 ratio) treatment; (B) effects of varying concentrations of RSV on cell viability; (C) cell viability under combined OA/PA and RSV treatment; (D) oil red O staining of lipid accumulation in OA/PA-induced cells; (E) quantitative analysis of red oil red O-stained areas; (F) absorbance measurements at 510 nm for oil red O staining; (G) serum TC levels under combined treatment; (H) serum TG levels under combined treatment. Quantitative analysis of each band values shown are means ± SD, ***p < 0.001 *versus* control; **p < 0.01 *vs.* control, *p < 0.05 *vs.* control, ##*p* < 0.01 *vs.* model, #*p* < 0.05 *vs.* model, *n* = 6.

The selection of appropriate adipocyte-inducing agents is vital for anti-steatosis research, and the OA/PA complex has extensively been adopted owing to its ability to mimic physiological lipotoxic microenvironments. In this study, a HepG2 cell steatosis model was established *via* OA/PA (2 : 1 ratio) induction to systematically evaluate the regulatory effects of RSV on lipid metabolism. As shown in Fig. 3A, OA/PA treatment significantly inhibited HepG2 cell viability in a concentration-dependent manner (cell viability decreased to 37.47% at 2000 μM). RSV, a classical polyphenolic anti-inflammatory molecule, has previously been revealed to ameliorate hepatic lipid metabolism disorders through multi-target mechanisms.^38,39^ In RSV monotherapy experiments (Fig. 3B), low concentrations of RSV (1–10 μM) exhibited mild pro-proliferative effects (cell viability remained at ∼100%), while higher concentrations (>10 μM) diminished viability, indicating a dose-dependent hormetic effect on RSV-mediated cytoprotection.

Based on these findings, we further investigated RSV's therapeutic potential against OA/PA (500 μM)-induced cytotoxicity and steatosis. Combination treatment experiments (Fig. 3C) revealed that low-dose RSV (1–10 μM) significantly alleviated OA/PA-induced cell viability suppression, suggesting that RSV potentially exerts protective effects by counteracting lipotoxic stress. To validate this hypothesis, intracellular lipid accumulation was assessed *via* oil red O staining. As shown in Fig. 3D, RSV treatment markedly reduced lipid droplet number and size. Quantitative analysis of stained red areas (Fig. 3E) and absorbance measurements (Fig. 3F) further confirmed an approximately 40% reduction in lipid deposition (*p* < 0.01). Additionally, co-treatment with RSV and OA/PA significantly downregulated TG (52.1% reduction) and TC (40.6% reduction) levels in the culture medium (Fig. 3G–H), indicating improved lipid transport and metabolism in lipotoxic microenvironments.

These results demonstrate that RSV exhibits dual regulatory properties in OA/PA-induced HepG2 cells, maintaining cell viability while suppressing lipid accumulation and mitigating metabolic dysregulation. However, RSV's cytoprotective efficacy was inferior to that of the classic hepatoprotective agent silymarin, possibly because of its limited cell membrane permeability and insufficient intracellular effective concentrations. Considering that LNPs can significantly enhance drug bioavailability through ApoE-mediated hepatic targeting,^40^ subsequent experiments employed LNP encapsulation to optimise RSV delivery efficiency, providing novel insights into NAFLD therapy.

### 3.4 Preparation, characterisation, and intracellular delivery efficiency of LNPs for drug delivery

**Fig. 4:**
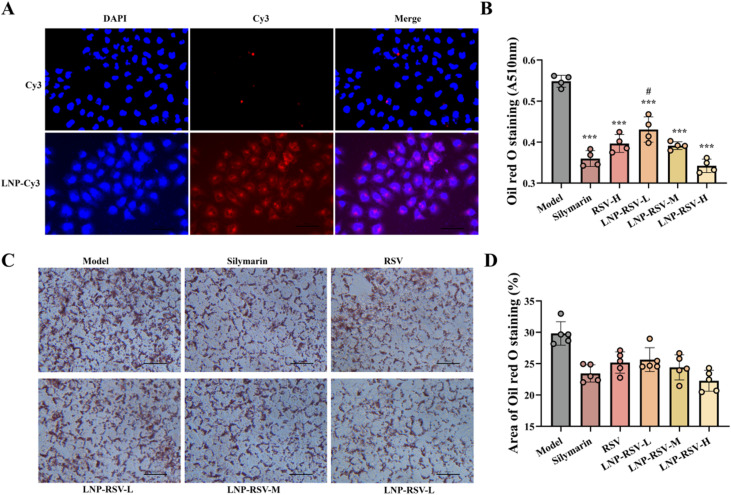
(A) Schematic diagram of the cellular uptake of LNP-Cy3; (B)Absorbance measurements at 510 nm for oil red O staining; (C) oil red O staining absorbance measurements of LNP-RSV-treated cells; (D) quantitative analysis of red oil red O-stained areas. Quantitative analysis of each band values shown are means ± SD, ***p < 0.001 *versus* model; #*p* < 0.05 *vs.* silymarin, *n* = 6.

In experiments investigating the impact of LNPs on the hepatic delivery of RSV, we co-encapsulated fluorescent probes (Cy3) with LNPs. The results demonstrated that LNP-Cy3 exhibited significantly enhanced cellular uptake compared with free Cy3 (Fig. 4A), indicating improved biocompatibility and absorption efficiency *via* LNP-mediated delivery. However, consistent with prior reports emphasizing the limited active targeting capability of conventional LNPs,^41^ LNP-encapsulated RSV (LNP-RSV) merely displayed modest anti-steatotic efficacy—superior to that of free RSV but comparable to that of silymarin—in lipid-laden HepG2 cells (Fig. 4B–D).

### 3.5 Role of Gal-modified LNPs in cellular uptake and hepatic-targeted drug distribution

To further evaluate the biological properties of Gal-LNPs, this study systematically investigated their cytocompatibility, delivery efficiency, and mechanisms of action. Cytotoxicity assays demonstrated that Gal-LNPs exhibited no significant dose-dependent toxicity toward HepG2 cells within the concentration range of 0–1 mg mL^−1^, indicating favourable biosafety.

**Fig. 5:**
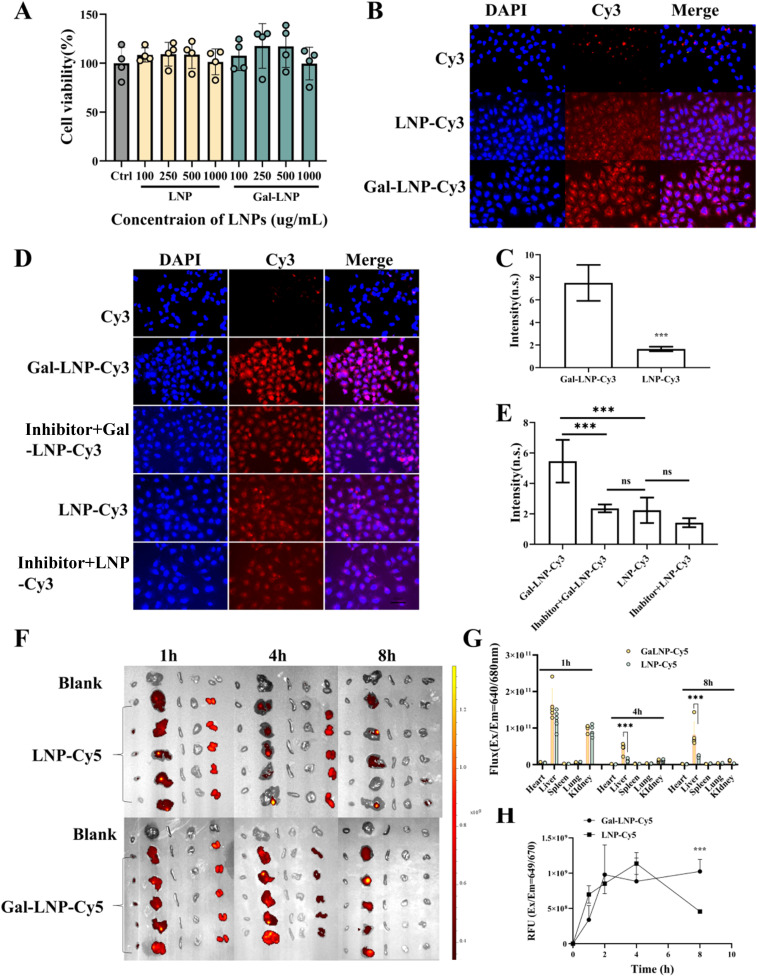
(A) Cell viability assessment following Gal-LNP treatment at total doses (100–1000 μg mL^−1^); (B) cellular uptake images of LNP-Cy3 *versus* Gal-LNP-Cy3; (C) quantitative analysis of LNP-Cy3 and Gal-LNP-Cy3 cellular uptake; (D) effect of Gal inhibitor on Gal-LNP-Cy3 uptake; (E) quantitative analysis of Gal-LNP-Cy3 uptake post-Gal inhibitor treatment; (F) IVIS imaging of drug distribution at various time points after tail vein injection of LNP-Cy3 and Gal-LNP-Cy3 in mice; (G) quantitative analysis of hepatic fluorescence intensity post-injection; (H) quantitative analysis of serum fluorescence intensity post-injection. Quantitative analysisof each band. Values shown are means ± SD, ***p < 0.001, *n* = 5.

To visually compare drug internalisation efficiency between delivery systems, Cy3-labelled LNP-Cy3 and Gal-LNP-Cy3 were constructed. Fluorescence microscopy (Fig. 5B) and quantitative analysis (Fig. 5C) revealed a 3.5-fold increase (*p* < 0.001) in fluorescence intensity for Gal-LNP-Cy3-treated cells compared with that for unmodified LNP-Cy3-treated cells, suggesting Gal modification significantly enhanced cellular uptake. To elucidate the underlying molecular mechanism, cells were pre-treated with a Gal competitive inhibitor (50 mM). As shown in Fig. 5C and D, inhibitor treatment reduced Gal-LNP-Cy3 fluorescence intensity by 56.7% (*p* < 0.01), while LNP-Cy3 merely exhibited an 11.2% decrease (*p* > 0.05). These findings align with recent research advancements in Gal-modified LNP. Sato A *et al.* demonstrated that Gal-modified cationic LNP significantly enhance nucleic acid delivery efficacy^42^ and this delivery enhancement pattern was similarly observed with small-molecule drug.^35^ Furthermore, in Nie's investigation, ASGPR binding affinity using multiple Gal-based ligands was systematically evaluated, demonstrating significantly higher Gal/GalNAc-mediated cellular internalization compared to unmodified controls, mechanistically confirming that cellular uptake of Gal-LNPs depends on ASGPR-mediated endocytosis.^43^

*In vivo* targeting performance was further assessed *via* small-animal live imaging. Following tail vein injection with Cy5-labelled Gal-LNP-Cy5, IVIS imaging revealed a hepatic fluorescence intensity peak at 4 h, representing a 3.9-fold enhancement over unmodified LNP-Cy5 (Fig. 5E and F). These results demonstrate that Gal modification optimises LNP performance regarding intracellular delivery and hepatic targeting, providing a novel strategy for liver-directed delivery systems. Recent investigations have also well-proved the enhanced hepatic targeting capacity of Gal-modified LNPs *in vivo*. As evidenced by Ren *et al.*, surface galactosylation of LNPs significantly improves their uptaked efficiency by hepatocytes and *in vivo* liver targeting enrichment.^44^ Notably, our study revealed that at 8 hour post-administration, the Gal-LNP-CY5 formulation demonstrated substantially higher serum fluorescence intensity compared to its non-galactosylated counterpart (LNP-CY5). This observation suggests a dual mechanistic role of Gal modification: potential extension of systemic circulation half-life and possible augmentation of liver-targeting efficiency through optimized biodistribution patterns.

### 3.6 Validation of the intervention effect of Gal-LNP-RSV on OA/PA-induced steatosis *via* oil red O staining and lipid metabolism analysis

**Fig. 6:**
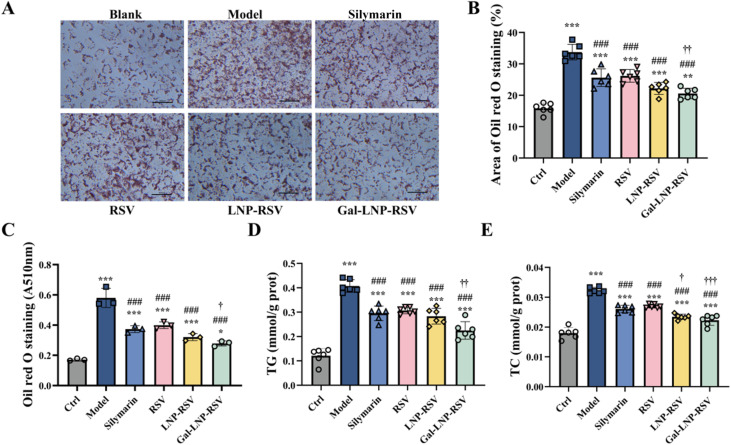
(A) Oil red O staining of lipid accumulation in OA/PA-induced HepG2 cells following drug intervention; (B) quantitative analysis of stained lipid areas; (C) absorbance measurement of oil red O at 510 nm; (D) TG levels; (E) TC levels. Quantitative analysis of each band. Values shown are means ± SD, ***p < 0.001 *versus* control; **p < 0.01 *vs.* control, *p < 0.05 *vs.* control, ##*p* < 0.01 *vs.* model, #*p* < 0.05 *vs.* model, †††*p* < 0.001 *vs.* silymarin, ††*p* < 0.01 *vs.* silymarin, †*p* < 0.05 *vs.* silymarin, *n* = 6.

To systematically evaluate the regulatory advantages of Gal-LNP-RSV in lipid metabolism, this study compared the therapeutic effects of different delivery systems at the optimal concentration (5 μM). Quantitative oil red O staining analysis (Fig. 6A and B) revealed that the lipid droplet area percentage in the Gal-LNP-RSV group (20.6%) was significantly lower than that in the LNP-RSV group (22.2%, *p* < 0.01), representing a significant reduction compared with that in the model group (62.4%) (Fig. 6C). Lipid metabolic parameter analysis further confirmed that the TG level in the Gal-LNP-RSV group (0.225 mmol per g prot) was lower than that in the LNP-RSV group (0.283 mmol per g prot, *p* < 0.05), while its TC level (0.0223 mmol per g prot) significantly decreased compared with that of the LNP-RSV group (0.0235 mmol per g prot, *p* < 0.01) (Fig. 6D and E).

These results demonstrate that Gal-LNP-RSV exhibits significantly superior regulatory effects on ameliorating lipid metabolic disorders compared with LNP-RSV, suggesting that its enhanced therapeutic efficacy may stem from improved targeting efficiency.

### 3.7 Validation of the therapeutic efficacy of Gal-LNP-RSV in NAFLD using animal experiments

**Fig. 7:**
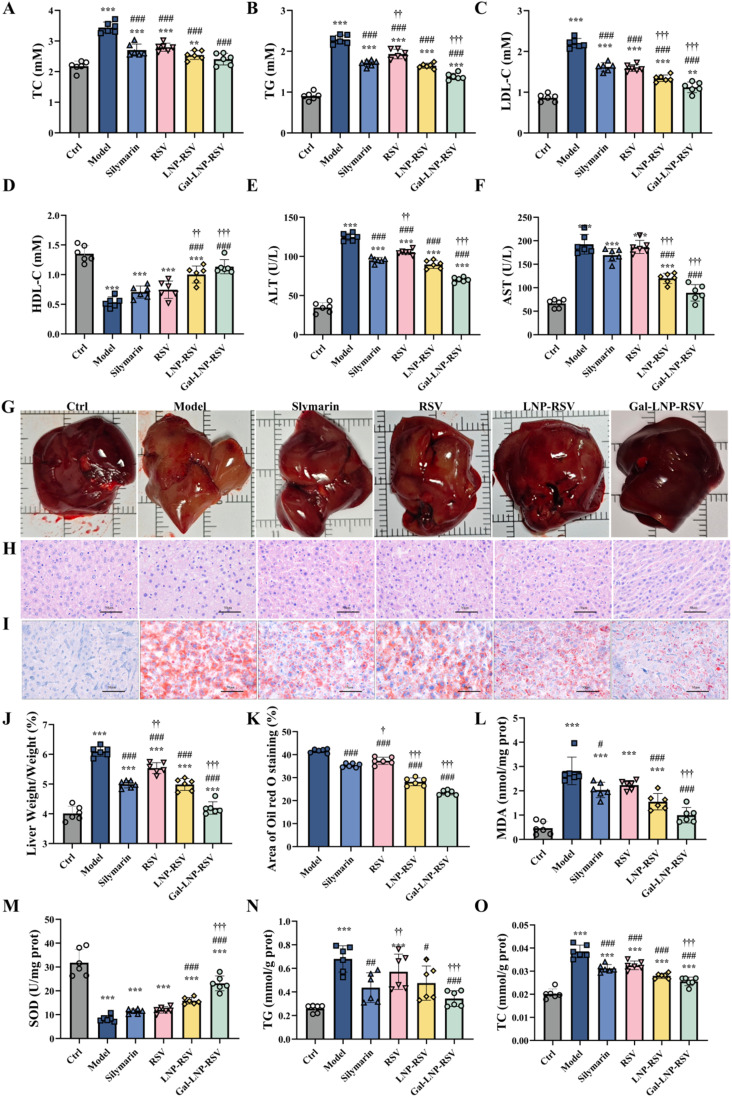
(A) Serum TC; (B) serum TG; (C) serum LDL-C; (D) serum HDL-C; (E) serum ALT; (F) serum AST; (G) macropathological observation of liver tissue; (H) hepatic H&E staining; (I) hepatic oil red O staining; (J) liver weight changes; (K) quantitative analysis of hepatic lipid deposition area; (L) hepatic superoxide dismutase (SOD) activity; (M) hepatic MDA content; (N) hepatic TG content; (O) hepatic TC content. Quantitative analysis of each band. Values shown are means ± SD, ***p < 0.001 *versus* control; **p < 0.01 *vs.* control, *p < 0.05 *vs.* control, ##*p* < 0.01 *vs.* model, #*p* < 0.05 *vs.* model, †††*p* < 0.001 *vs.* silymarin, ††*p* < 0.01 *vs.* silymarin, †*p* < 0.05 *vs.* silymarin, *n* = 6.

Based on the biodistribution characteristics and preliminary biochemical findings, this study systematically evaluated the therapeutic effects of Gal-LNP-RSV in an HFD-induced NAFLD mouse model. A 6-week HFD intervention successfully established NAFLD, with the model group exhibiting significant lipid metabolism disorders (serum TC, TG, and LDL-C levels increased 1.58–2.54-fold compared with those in the control group, while HDL-C levels significantly decreased) and typical hepatic steatosis phenotypes (Fig. 7G). H&E staining revealed disrupted hepatic lobular architecture with intracellular lipid droplet infiltration, and oil red O staining confirmed extensive lipid droplet formation in liver tissue.

We treated NAFLD mice with silymarin (positive control), free RSV, LNP-RSV, and Gal-LNP-RSV. All treatments improved lipid metabolism, liver function, and oxidative stress to varying degrees. However, free RSV displayed inferior efficacy to silymarin, whereas LNP-RSV exhibited comparable or slightly superior effects to silymarin, consistent with cellular experimental results.

Among all treatments, Gal-LNP-RSV demonstrated the most pronounced therapeutic outcomes. In lipid metabolism and hepatic dysfunction regulation, Gal-LNP-RSV significantly deregulated serum TG, LDL-C, AST, and ALT compared with LNP-RSV, with improvements ranging from approximately 13% to 26%. Additionally, Gal-LNP-RSV restored the liver-to-body weight ratio to baseline levels (Fig. 7D) and reduced hepatic lipid accumulation, TG content, and TC content by 43.6%, 49.1%, and 33.5%, respectively, compared with the NAFLD model (*p* < 0.05), outperforming LNP-RSV (32.3%, 29.8%, and 27.2%, respectively) (Fig. 7H–I). In the model group, SOD activity decreased to 73.3% that of the control group, while MDA levels surged by 753.0%. Gal-LNP-RSV treatment restored SOD activity to 74.2% of the normal level and reduced MDA content to 273% that of the control (Fig. 7F and G), markedly surpassing LNP-RSV's effects (50.7% SOD activity and 462.5% MDA content, *p* < 0.05).

Previous investigations have established Gal-LNP systems as effective carriers for hepatic-targeted delivery of both nucleic acids and small-molecule drugs in treating various liver disorders.^45^ The incorporation of galactose (Gal) modifications has substantially augmented the therapeutic value of LNPs in hepatology by enhancing cellular internalization efficiency and delivery precision, thereby optimizing treatment outcomes. To date, four GalNAc-conjugated siRNA therapeutics have reached clinical application, with Alnylam spearheading their development and commercialization.^46^ These collective advancements unequivocally demonstrate the exceptional potential of Gal-LNP platforms in revolutionizing hepatic disease management.

This study is the first to employ a multimodal evaluation system to elucidate Gal-LNP-RSV's multi-dimensional protective mechanisms against NAFLD, reversing steatosis at the anatomical level, correcting lipid imbalance metabolically, and restoring redox homeostasis molecularly. These findings provide crucial experimental evidence for developing natural-product-based anti-steatotic formulations *via* targeted delivery systems, further validating the superiority of the Gal-LNP platform.

## 4 Conclusion

This study successfully developed a Gal-modified LNP system (Gal-LNP-RSV) for targeted NAFLD therapy. *In vitro* and *vivo* experiments demonstrated that Gal-LNP significantly enhances hepatic and intracellular drug delivery efficiency compared with unmodified LNPs. In lipid-laden HepG2 cells and HFD-induced NAFLD mouse models, Gal-LNP-RSV exhibited superior therapeutic efficacy, effectively reducing hepatic lipid accumulation, restoring serum ALT/AST levels, and ameliorating oxidative stress. The optimised formulation leverages ASGPR-mediated active targeting through its Gal-PEG-DSPE component, synthesised *via* a clinically scalable one-step conjugation strategy. These findings provide indispensable experimental evidence for advancing natural product-based anti-steatotic formulations using a rational nanocarrier design. This study substantiates that Gal-modified LNP can enhance liver-targeted delivery efficiency of drugs, yet critical limitations persist. Such as, current modification strategies demonstrate incomplete differentiation in targeting specificity between hepatocytes and non-parenchymal liver cells, potentially compromising therapeutic precision; additionally, whether the differences in ASGPR expression on hepatocytes at different disease stages simultaneously affect the enrichment and delivery efficiency of GAL-LNP; the evidents of dose-effect relationship between Gal density and targeting efficiency is still limited. Such issues will be explored in future studies to bridge the gap from preclinical research to clinical translation.

## Author contributions

Zhijie Liang: methodology, investigation, formal analysis, writing – original draft; Jinzhuai Li: methodology, formal analysis, writing – original draft, investigation; Shuying Luo: writing – original draft, investigation; Shaorong Li: writing – original draft; Kun Zhao: funding acquisition, data curation, investigation; Hongmian Jiang: funding acquisition, investigation; Yisi Ou: resources, investigation; Juan Zhong: investigation; Lifeng Luo: investigation; Lihua Huang: conceptualization, writing – review & editing, resources; Yingying Li: conceptualization, supervision, writing – review & editing, resources.

## Conflicts of interest

The authors declare that there are no conflicts of interest.

## Supplementary Material

RA-015-D5RA02554K-s001

## Data Availability

The data used in this study can be requested *via* the email of the corresponding authors. Requesters are required to provide relevant information to facilitate the verification and processing of the access request. We encourage interested researchers to contact us for data access, ensuring compliance with applicable ethical and legal regulations.
