# Supplementary material for: Galactose receptor-mediated hepatic targeting system: engineering of quinary cationic liposomes for resveratrol delivery against hepatic steatosis
Source: RSC Adv. 2025 Jun 11;15(25):19786–801. doi: 10.1039/d5ra02554k (PMC12153050; doi:10.1039/d5ra02554k)
Supplement: RA-015-D5RA02554K-s001 [file RA-015-D5RA02554K-s001.pdf]

Supplementary Information (SI) for RSC Advances.
This journal is © The Royal Society of Chemistry 2025

Galactose Receptor-Mediated Hepatic Targeting System: Engineering of Quinary Cationic Liposomes for Resveratrol Delivery Against Hepatic Steatosis

Zhijie Liang[1,2#] Jinzhuai Li[3#] Shuying Luo[2] Shaorong Li[2] Kun Zhao[4] Hongmian Jiang[2] Yisi Ou[5] Juan Zhong[2] Lifeng Luo[2] Lihua Huang[2*] Yingying Li[5*]

1 Shenzhen Research Institute of Lanzhou University, Shenzhen 518000, China.

2 Medical experimental center, The Fifth Affiliated Hospital of Guangxi Medical Universityl, Nanning 530000, China.

3 State Key Laboratory for the Chemistry and Molecular Engineering of Medicinal Resources, School of Chemistry and Pharmaceutical Sciences, Guangxi Normal University, Guilin 541004, China.

4 Departments of Hepatobiliary Surgery, The First Affiliated Hospital of Guangxi Medical University, Nanning 530021, China.

5 Department of traditional Chinese medicine,The Reproductive hospital of Guangxi Zhuang Autonomous Region, Nanning 530000, China.

#These authors contributed equally to this work.

*Corresponding authors:

Lihua Huang, Email: hualihuang999@126.com

Yingying Li, Email: lyynaturemed@163.com

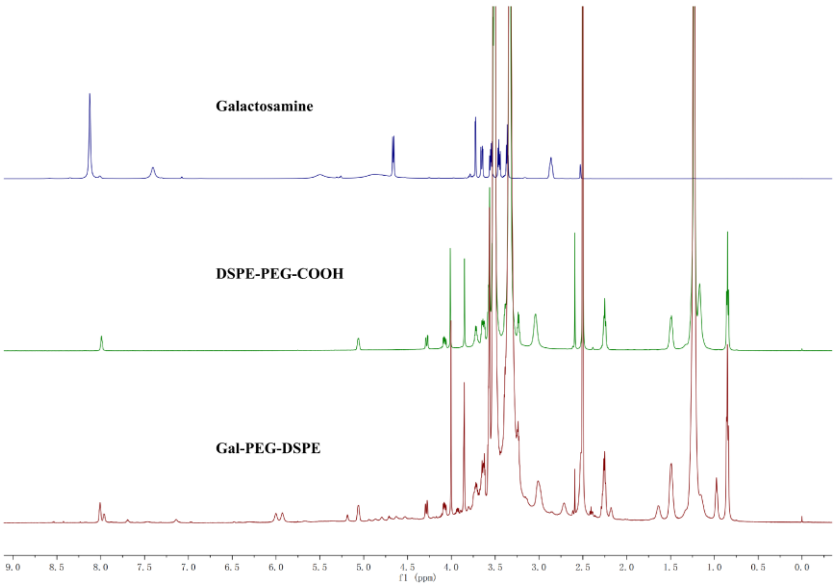

Figure S1. ^{1}H NMR spectra of Galactosamine、DSPE-PEG-COOH and Gal-PEG-DSPE.

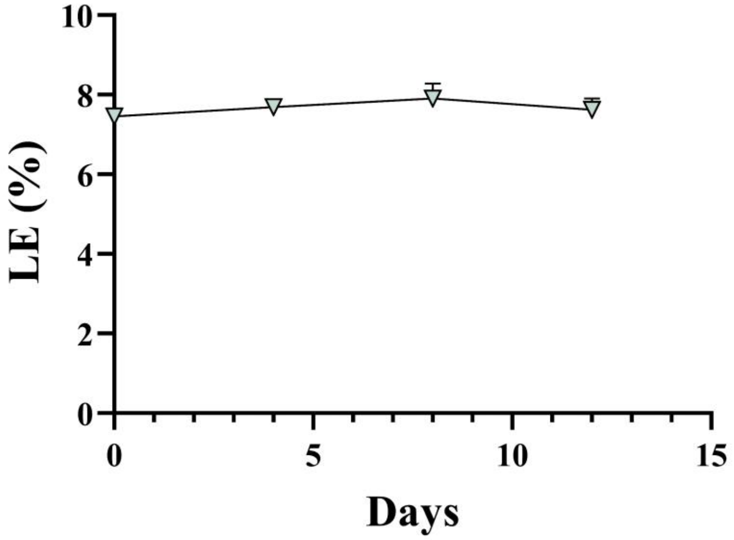

Figure S2. Schematic Diagram of Loading Efficiency (LE%).

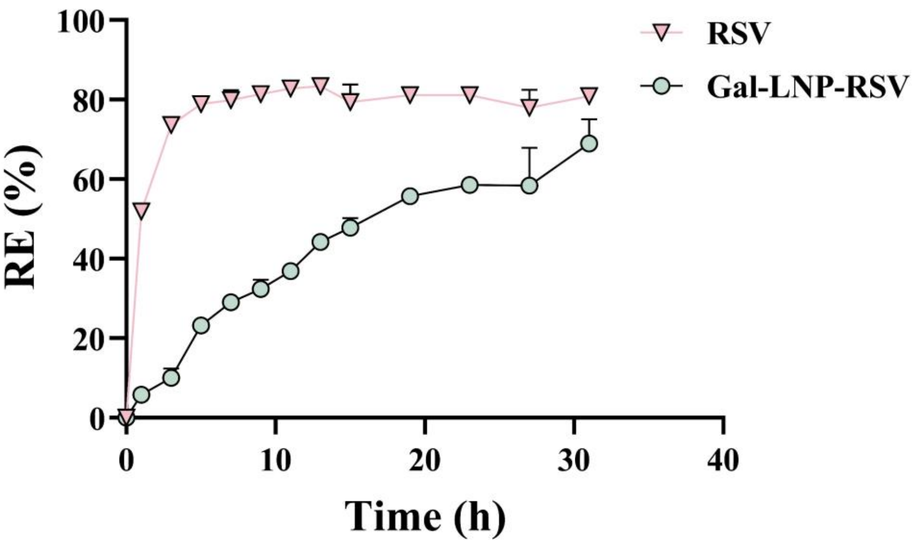

Figure S3. In Vitro Release Efficiency (RE%).

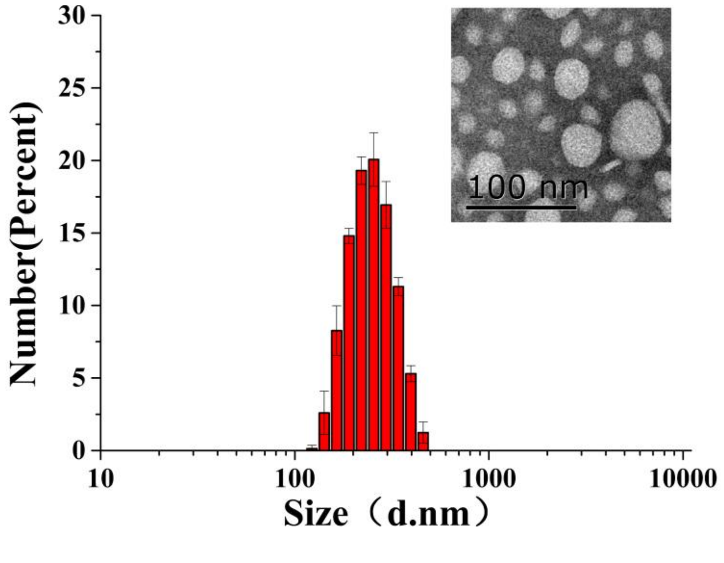

Figure S4. Particle size and TEM images of Gal-LNPs.

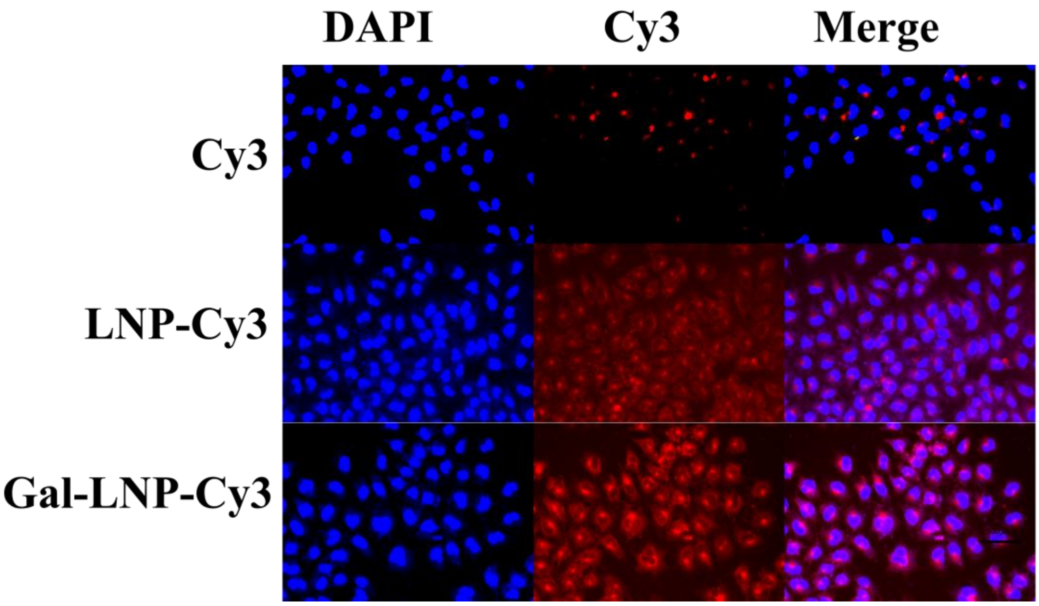

Figure S5. Cellular uptake images of LNP-Cy3 versus Gal-LNP-Cy3.

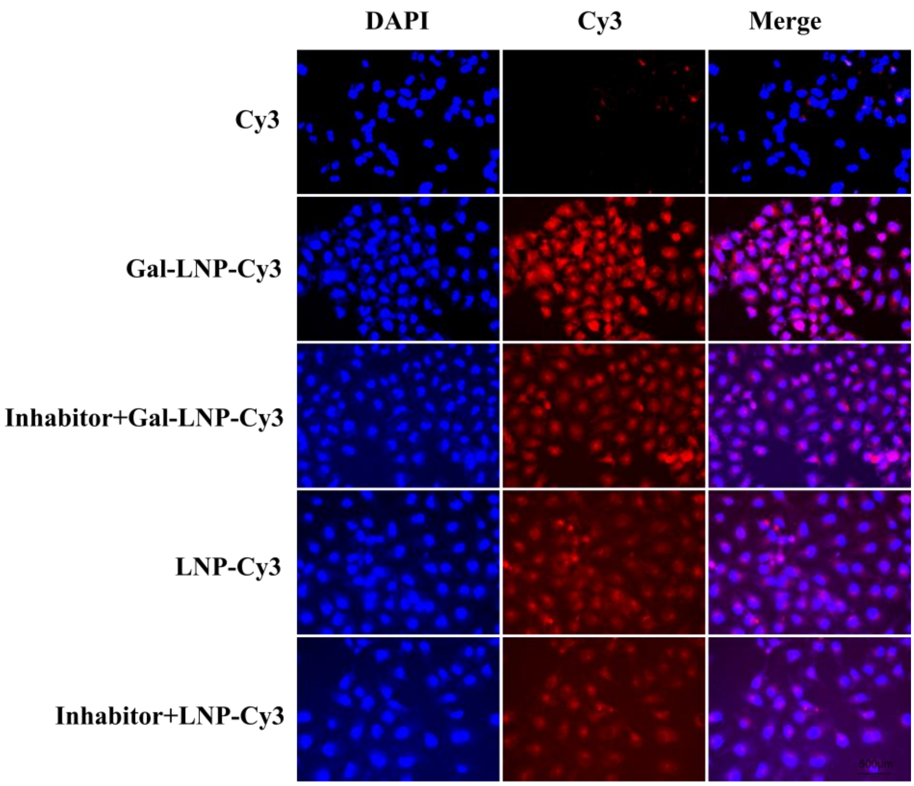

Figure S6. Effect of Gal inhibitor on Gal-LNP-Cy3 uptake.

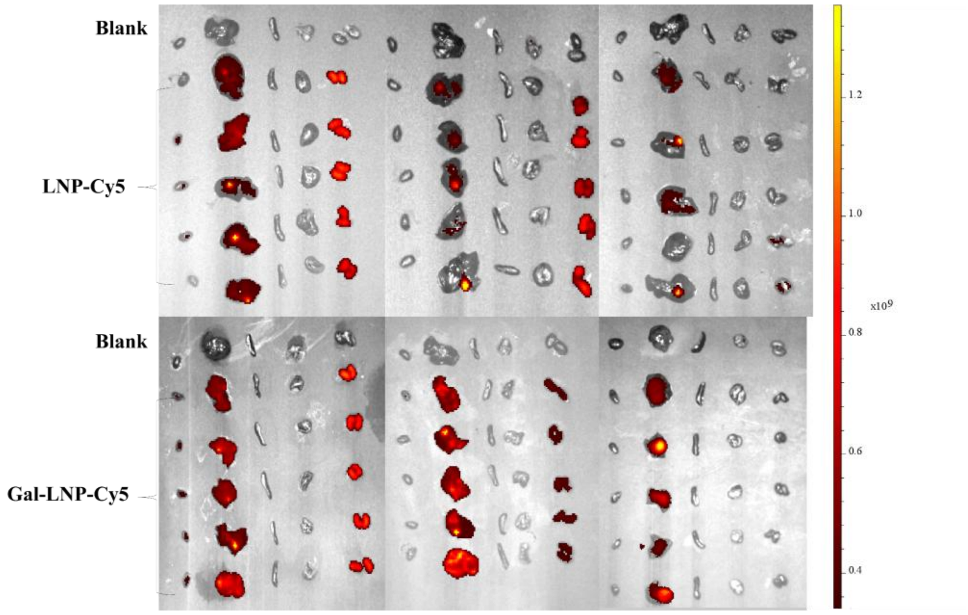

Figure S7. IVIS imaging of drug distribution at various time points after tail vein injection of LNP-Cy3 and Gal-LNP-Cy3 in mice.
